# Clot Without a Picture: Treating Massive PE When the CT Can’t See You Now

**DOI:** 10.51894/001c.166029

**Published:** 2026-08-01

**Authors:** Alaaeddine Talab, Christopher Nedzlek, Adam Hafeez, Michael Reaume

**Affiliations:** 1 Henry Ford - Wyandotte

## 128

### Introduction

Massive pulmonary embolism (PE) carries significant mortality and requires rapid reperfusion. CT pulmonary angiography (CTPA) is the diagnostic gold standard, however, hemodynamic instability may preclude safe imaging. Clinicians must then integrate clinical gestalt, risk stratification, and multidisciplinary judgment to guide empiric treatment. We present a case in which clinical probability alone justified systemic thrombolysis in a patient with refractory hypoxemia unable to undergo confirmatory imaging.

### Case Description

A 75-year-old male with coronary artery disease, hypertension, sick sinus syndrome with pacemaker, and recent left lower extremity tendon repair presented with acute dyspnea and pleuritic chest pain of six hours’ duration. EMS documented oxygen saturation of 78% on room air. Saturations remained below 83% despite high-flow nasal cannula and noninvasive ventilation at 100% FiO₂. The patient declined intubation and reaffirmed DNR/DNI status after shared decision-making.

Chest radiography showed clear lung fields. ECG demonstrated a paced rhythm not meeting Sgarbossa criteria. Troponin elevation was attributed to right ventricular strain rather than primary ACS. The clinical picture, profound refractory hypoxemia, tachycardia, recent immobilizing surgery, no infectious or mechanical etiology, and failure of maximal respiratory support, created a compelling gestalt for massive PE with right heart strain.

Persistent instability precluded safe CTPA. Bedside consultation between emergency medicine, cardiology, and the ICU was performed. Given high clinical probability and no safe alternatives, empiric systemic thrombolysis with tPA was administered prior to imaging confirmation.

Following thrombolysis, saturation improved from the low 80s to 92% on BiPAP, then 100%. CTPA confirmed bilateral pulmonary emboli with an RV/LV ratio of 1.9. Lower extremity Doppler revealed an occlusive soleal vein DVT. Echocardiography demonstrated severe RV enlargement and McConnell’s sign. The patient was anticoagulated and discharged in stable condition.

### Discussion

This case demonstrates that empiric thrombolysis without imaging can be lifesaving when massive PE probability is high and CTPA is unobtainable. Key decision drivers included refractory hypoxemia unresponsive to maximal noninvasive support, a clear provoked risk factor, exclusion of competing diagnoses, and real-time multidisciplinary consensus. Positive-pressure ventilation was deliberately avoided given risk of worsening right ventricular failure. This case reinforces structured clinical gestalt and bedside collaboration in time-critical emergencies.

